# Depth of response is a significant predictor for long-term outcome in advanced gastric cancer patients treated with trastuzumab

**DOI:** 10.18632/oncotarget.16099

**Published:** 2017-03-10

**Authors:** Choong-Kun Lee, Seung-Seob Kim, Saemi Park, Chan Kim, Su Jin Heo, Joon Seok Lim, Hyunki Kim, Hyo Song Kim, Sun Young Rha, Hyun Cheol Chung, Sohee Park, Minkyu Jung

**Affiliations:** ^1^ Division of Medical Oncology, Department of Internal Medicine, Yonsei Cancer Center, Yonsei University, College of Medicine, Seoul, Korea; ^2^ Department of Radiology, Yonsei University College of Medicine, Seoul, Korea; ^3^ Department of Biostatistics, Graduate School of Public Health, Yonsei University, Seoul, Korea; ^4^ Department of Pathology, Yonsei University College of Medicine, Seoul, Korea

**Keywords:** gastric cancer, trastuzumab, depth of response, early tumor shrinkage, survival

## Abstract

**Purpose:**

We aimed to determine and compare the predictive values of depth of response (DpR) and early tumor shrinkage (ETS) on long-term outcomes in gastric cancer patients treated with trastuzumab.

**Results:**

From a total of 368 computed tomography examinations, DpR and ETS were evaluated. DpR was a significant tumor-size metric in predicting PFS and OS, and showed better discriminatory ability (higher Cτ indices, 0.6957 for PFS; 0.7191 for OS) than ETS. DpR ≥ 45% (vs. < 45%) was the optimal cutoff value, as it was best able to identify patients with longer PFS (median 9.0 vs. 6.3 months, hazard ratio [HR] = 0.608; 95% confidence interval [CI]: 0.335 to 1.104; *P* = 0.102) and OS (median 23.5 vs. 13.1 months, HR = 0.441; 95% CI: 0.203 to 0.955; *P* = 0.038).

**Materials and Methods:**

Sixty-one gastric cancer patients who received first-line trastuzumab-based chemotherapy were assessed for DpR and ETS. We employed Kaplan-Meier estimates, log-rank tests, Cox proportional hazards regression models, time-dependent receiver operating characteristics, and Youden's *J* index to evaluate and determine cutoff values of DpR and ETS as predictors of progression-free survival (PFS) and overall survival (OS).

**Conclusions:**

DpR and ETS were significant predictors of long-term outcomes in gastric cancer patients treated with first-line trastuzumab. Validation in prospective trials with larger patient populations is needed.

## INTRODUCTION

Gastric cancer, the second leading cause of cancer-related deaths worldwide, remains a major health problem despite a global decrease in its incidence [1]. Currently, trastuzumab-based first-line treatments represent the standard approach for human epidermal growth factor receptor 2 (HER2)-positive advanced gastric cancer (AGC) patients [2]; however, it is not equally effective in all such patients. Although identifying patients who will most benefit from this treatment is paramount, clinically applicable surrogate markers for survival have not been properly evaluated in AGC patients treated with trastuzumab. Even as *HER2* gene amplification was reported to predict overall survival (OS) in AGC patients undergoing trastuzumab-based chemotherapy [3], *HER2* gene amplification is not usually tested in patients who receive a score of 3+ on HER2 immunohistochemistry (IHC).

Defining appropriate surrogate end-points that predict long-term survival in prospective studies is important in the field of oncology. Aside from well-known surrogate end-points for OS, such as progression-free survival (PFS) and objective response rate, other metrics based on tumor size have recently been investigated. These include early tumor shrinkage (ETS) and depth of response (DpR), both of which are indicators of the percentage change in tumor size at a designated time point from the baseline. ETS, defined as change in tumor size at the time of first response evaluation, has been proposed to be associated with long-term outcomes in various cancer types and regimens, mainly colorectal cancer [4–10]. Recently, the concept of DpR [11], defined as the maximal tumor shrinkage observed [12], has gained attention in colorectal cancer patients treated with cetuximab or bevacizumab. Increased DpR was observed to significantly correlate with post-progression survival (PPS) or OS in cetuximab- or bevacizumab-treated colorectal cancer patients in large phase III trials [11, 13, 14]. Analysis of tumor-size metrics in association with survival has never been performed for gastric cancer. HER2-positive gastric cancers, which usually metastasize to measurable lesions in lymph nodes or the liver [3, 15] are an ideal system in which to test the usage of DpR or ETS as surrogate markers for long-term outcome. Additionally, while there have been separate studies on the association of DpR or ETS and cancer patient prognosis, there has been no study evaluating DpR or ETS as predictive factors for survival outcome within one treatment cohort, particularly in gastric cancer patients.

The purpose of this study was to investigate the predictive values of DpR and ETS on long-term outcomes in AGC patients treated with trastuzumab-based chemotherapy. We also sought to perform our investigation by using tumor-size metrics as surrogate markers and defining discriminatory cutoff values for dichotomizing patient populations according to DpR or ETS to guide treatment planning.

## RESULTS

### Patients

A total of 61 of 88 gastric cancer patients treated with trastuzumab were eligible for this study (Supplementary Figure 1). Patients underwent trastuzumab treatment between December 2005 and March 2014, with a median follow-up duration of 24.3 months (interquartile range, 18.3 to 28.3 months). The main clinicopathological features and response data are shown in Table 1. *HER2/CEP17* ratio by SISH or FISH was categorized into two groups using a cutoff of 4.7, a previously reported cutoff value that predicted response and survival [3]. The median number of trastuzumab cycles administered was eight (range, 2 to 38).

**Table 1:** Clinicopathologic features and survival outcomes of patients (N = 61)

Characteristics	N	%
**Age**		
Median	60	
Range	33–80	
**Sex**		
Male	40	65.6
Female	21	34.4
**ECOG performance status**		
0	31	50.8
1	22	36.1
2	8	13.1
**WHO classification**		
Adenocarcinoma well differentiated	5	8.2
Adenocarcinoma moderately differentiated	37	60.7
Adenocarcinoma poorly differentiated	14	23
Signet ring cell	5	8.2
**Prior gastrectomy**		
Subtotal gastrectomy	8	13.1
Total gastrectomy	7	11.5
Not done	46	75.4
**Primary tumor location**		
Gastroesophageal junction	4	6.6
Cardia	10	16.4
Body/Antrum	47	77
**HER2 overexpression by IHC**		
2+	15	24.6
3+	46	75.4
***HER2/CEP17*** **ratio by SISH or FISH**		
2.0-4.69	11	18.0
≥ 4.7	11	18.0
Not determined	39	63.9
**Metastatic location**		
Lymph node	47	77.0
Liver	27	44.3
Peritoneum*	17	27.9
Lung	8	13.1
Bone	5	8.2
**Chemotherapy regimen**		
Herceptin + FP	9	14.8
Herceptin + XP	52	85.2
**Follow-up, months**		
Median	24.3	
Interquartile range	18.3–28.3	
**Progression-free survival, months**		
Events (Progression or Death)	54	88.5
Median	8.0	
95% CI	6.8–9.3	
**Overall survival, months**		
Events (Death)	35	57.4
Median	16.0	
95% CI	11.2–21.0	

Abbreviations: CI, confidence interval; ECOG, Eastern Cooperative Oncology Group; WHO, World Health Organization; HER2, human epidermal growth factor receptor 2; IHC, immunohistochemistry; CEP17, chromosome 17 centromere; SISH, silver in situ hybridization; FISH, fluoroscence in situ hybridization; FP, 5-FU+Cisplatin; XP, Xeloda+Cisplatin. *11 patients had measurable peritoneal seeding nodules.

### Comparing DpR with ETS in predicting long-term outcomes

**Figure 1:**
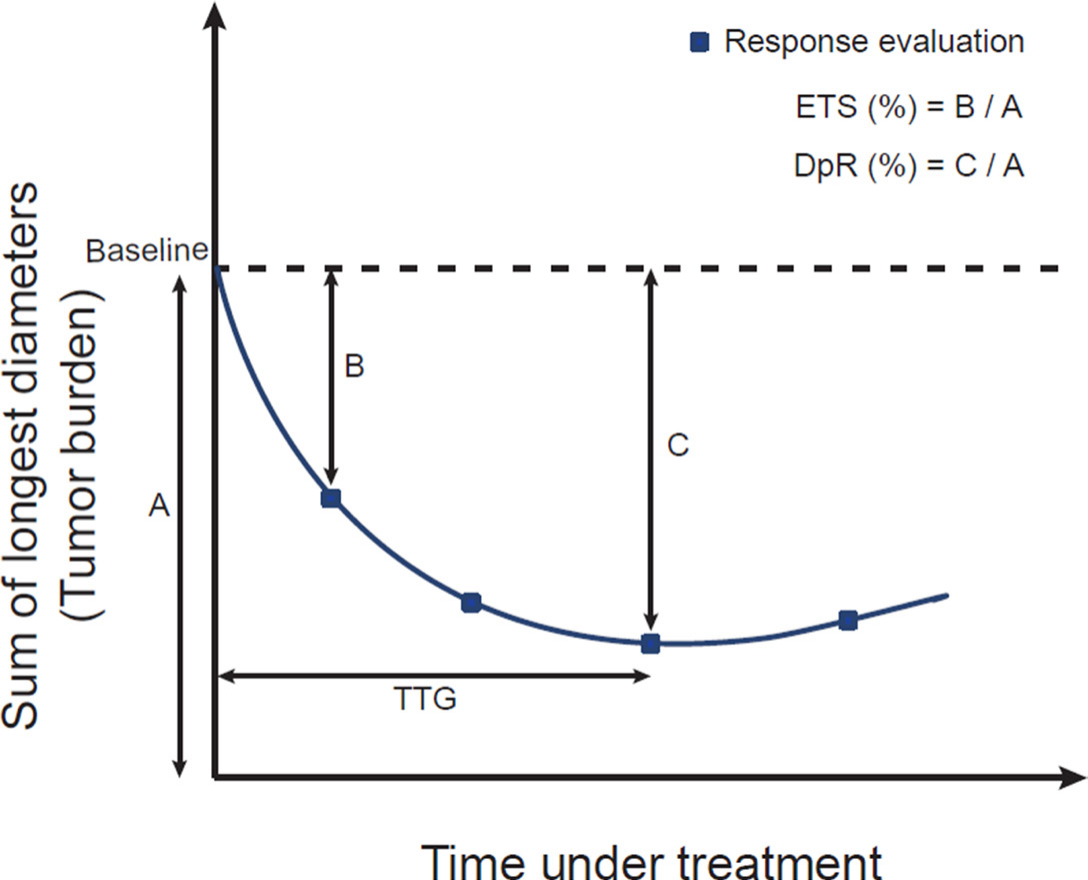
Schematic illustration for metrics of tumor size ETS, early tumor shrinkage; DpR, depth of response; TTG, time to tumor growth.

Relative tumor sizes compared to the baseline (the sum of the longest diameters according to RECIST) at the first evaluation (ETS) and at the best response (DpR) were evaluated from a total of 368 CT examinations (see Figure 1 for schematic illustration of tumor size metrics). The median time between the first trastuzumab administration and the first response evaluation (time to ETS) was 6 weeks (interquartile range, 5.3 to 7.1 weeks). In a univariate analysis, Eastern Cooperative Oncology Group (ECOG) performance status and HER2 overexpression as determined by IHC were significantly related to PFS and OS. Among the tumor size metrics, DpR was most strongly associated with both PFS and OS (*P* < 0.001), followed by time to tumor growth (TTG) and ETS (Supplementary Table 1). A multivariable Cox proportional hazards model for patient prognosis, adjusted for potential confounding factors including age, ECOG performance status, and HER2 overexpression, showed that DpR and ETS were significant predictors of long-term outcomes of both PFS and OS (Table 2 and Supplementary Table 2).

**Table 2:** Multivariable analysis of selected demographic variables

		Progression-free Survival			Overall Survival		
		Adjusted for Covariates			Adjusted for Covariates		
		HR	95% CI	*P*	HR	95% CI	*P*
Age	<65	Ref			Ref		
	≥ 65	0.777	0.423 to 1.426	0.415	1.001	0.482 to 2.069	0.997
ECOG performance status	0	Ref			Ref		
	≥ 1	1.746	0.967 to 3.153	0.065	2.284	1.090 to 4.785	**0.029**
HER2 overexpression by IHC	2+	Ref			Ref		
	3+	0.529	0.282 to 0.993	**0.048**	1.092	0.486 to 2.453	0.832
ETS^†^		0.858	0.765 to 0.961	**0.008**	0.869	0.758 to 0.996	**0.044**
Age	< 65	Ref			Ref		
	≥ 65	0.796	0.441 to 1.436	0.448	1.119	0.536 to 2.335	0.764
ECOG performance status	0	Ref			Ref		
	≥ 1	1.415	0.775 to 2.582	0.258	1.876	0.868 to 4.055	0.110
HER2 overexpression by IHC	2+	Ref			Ref		
	3+	0.626	0.333 to 1.179	0.147	1.616	0.671 to 3.891	0.284
DpR^†^		0.786	0.700 to 0.883	**< 0.0001**	0.798	0.698 to 0.912	**0.001**

†HR per 10% tumor size decrease

Abbreviations: HR, hazard ratio; CI, confidence interval; Ref, Reference; ECOG, Eastern Cooperative Oncology Group; HER2, human epidermal growth factor receptor 2; IHC, immunohistochemistry; ETS, early tumor shringkage; TTG, time to tumor growth; DpR, depth of response.

### Comparing the predictive accuracies of DpR and ETS as continuous variables for long-term outcomes

**Figure 2:**
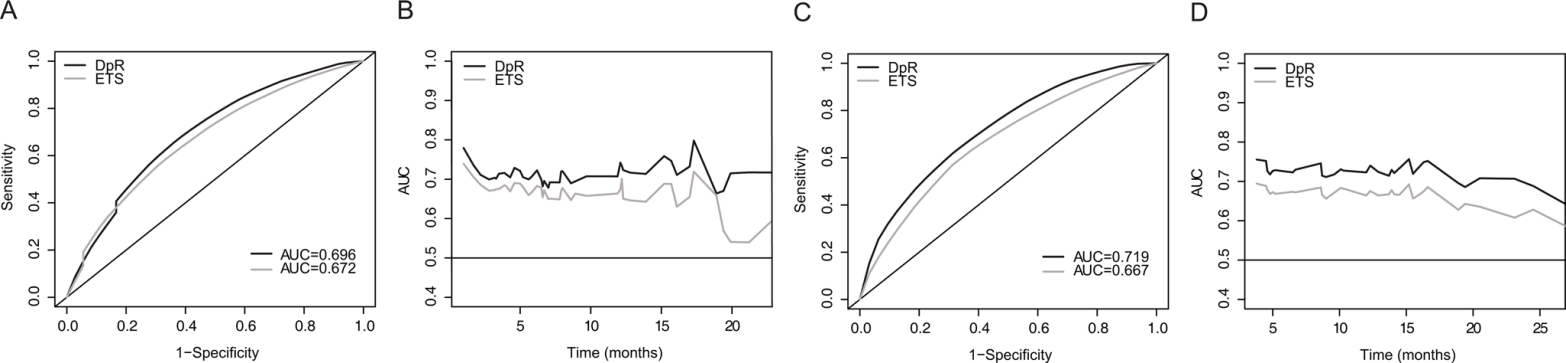
Time-dependent receiver operating characteristics curves of DpR and ETS for progression-free survival at 6.7 months* (**A**) and overall survival at 13.8 months* (**C**); time-dependent integrated AUC of DpR and ETS for progression-free survival (**B**) and overall survival (**D**). *6.7 months and 13.8 months were chosen from median progression-free survival and median overall survival data reported for the Trastuzumab for Gastric Cancer (ToGA) trial. AUC, area under the curve; ETS, early tumor shrinkage; DpR, depth of response.

Time-dependent receiver operating characteristics (TDROC) analysis was performed to compare the predictive accuracy of DpR and ETS as continuous variables for long-term outcomes (Table 3). DpR showed better discriminatory ability (higher C_τ_ indices, 0.6957 [95% CI: 0.6564 to 0.8176] for PFS; 0.7191 [95% CI: 0.6694 to 0.8465] for OS) than ETS (C_τ_ indices, 0.6722 [95% CI: 0.6131 to 0.7807] for PFS; 0.6681 [95% CI: 0.6040 to 0.7952] for OS). Higher C_τ_ indices were found for DpR than for ETS consistently for both PFS and OS over time (Figure 2). Time-specific accuracy decreased minimally over time for OS with both DpR and ETS (Figure 2D) yet appeared consistent in the TDROC curves of DpR and PFS (Figure 2B).

**Table 3:** Performance indices of early tumor shrinkage and depth of response on long-term outcome

	Progression-free Survival	Overall Survival
**ETS**		
Cτ index	0.6722	0.6681
95% CI^†^	0.6131 to 0.7807	0.6040 to 0.7952
Youden Index	0.2516	0.2573
Cutoff Value	29.3%	27.7%
**DpR**		
Cτ index	0.6957	0.7191
95% CI^†^	0.6564 to 0.8176	0.6694 to 0.8465
Youden Index	0.2943	0.3056
Cutoff Value	44.4%	43.3%

†95% CI was obtained from bootstrapping of 1000 samples.

Abbreviations: ETS, early tumor shrinkage; DpR, depth of response; Cτ, time-dependent concordance; CI, confidence interval.

### Clinical applicability of DpR and ETS

### Choice of cutoff values

The clinical utility of tumor-size metrics often involves dichotomization of the population around a cutoff threshold that best distinguishes the long-term outcomes between groups. In this analysis, we used Youden's *J* index, a commonly used measure of overall diagnostic effectiveness, to obtain the optimal cutoff points for both DpR and ETS. ROC curves of DpR and ETS depicting Youden's *J* index and optimal cutoff points for PFS or OS were constructed (Supplementary Figure 2). Cutoff values of 29.3% (PFS) and 27.7% (OS) for ETS and 44.4% (PFS) and 43.3% (OS) for DpR were obtained based on the highest sensitivity and specificity (Table 3). Cutoff values of 30% for ETS and 45% for DpR were chosen for further analyses.

### Long-term outcome prediction using DpR and ETS

**Figure 3:**
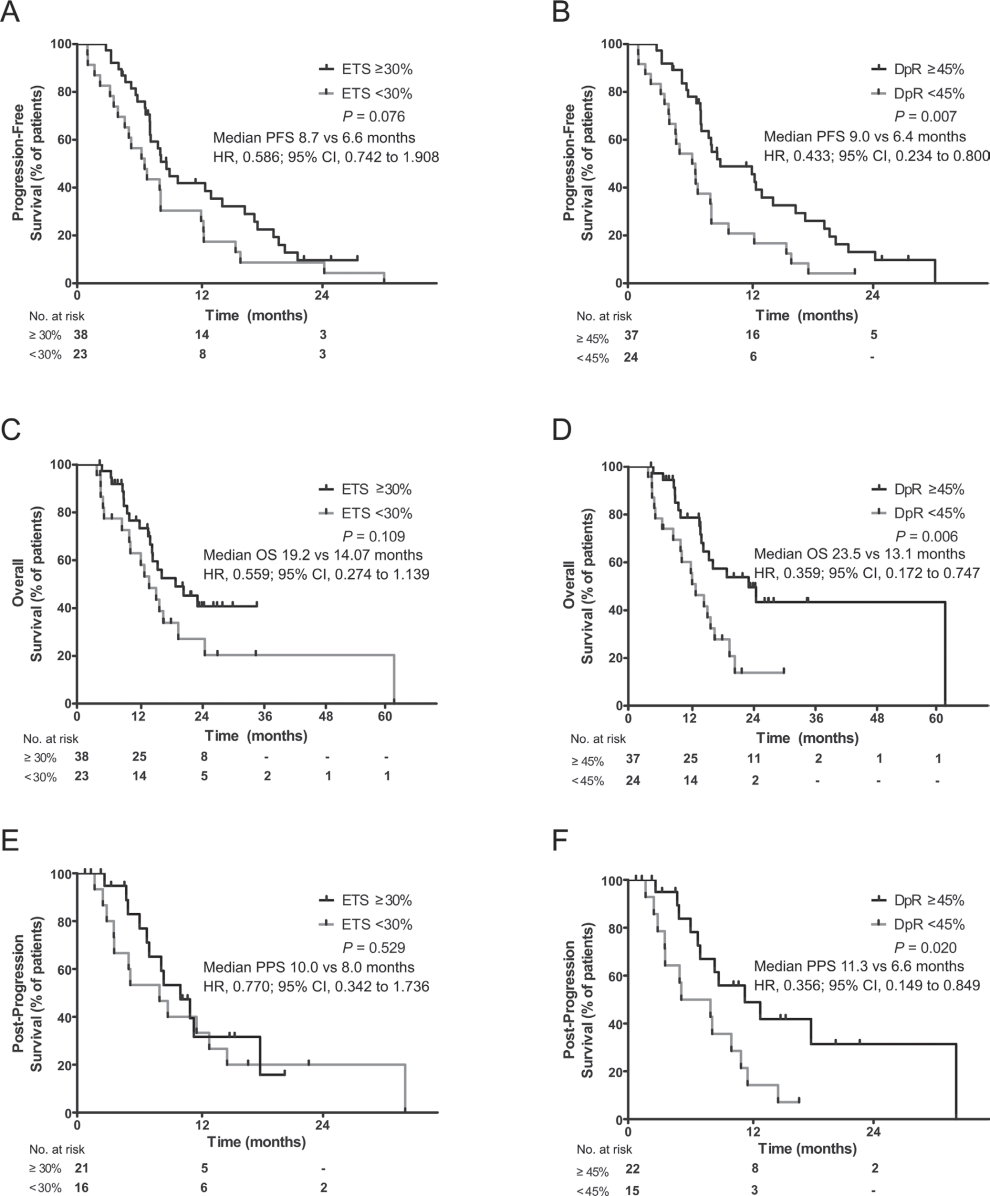
Kaplan-Meier curves for progression-free survival (**A, B**), overall survival (**C, D**), and post-progression survival (**E, F**) in relation to early tumor shrinkage (cutoff ≥ 30%; A, C, E) and depth of response (cutoff ≥ 45%; B, D, F). ETS, early tumor shrinkage; DpR, depth of response.

Patients were divided into subgroups according to cutoff values for ETS (< 30%: *N* = 23; ≥ 30%: *n* = 38) or DpR (< 45%: *N* = 24; ≥ 45%: *N* = 37), and their clinicopathological features and survivals were compared (Supplementary Table 3). Both groups had similar characteristics except for a better performance status and higher HER2 overexpression on IHC among the better response subgroups. PFS and OS were significantly longer for patients in the ≥ 45% DpR group yet not for those in the ≥ 30% ETS group. Kaplan-Meier plots for PFS and OS in relation to DpR and ETS, each categorized by their respective cutoff values, were also constructed (Figure 3). Dichotomized DpR was a significant predictor of PFS (*P* = 0.007) and OS (*P* = 0.006), although ETS was not (*P* = 0.076 for PFS; *P* = 0.109 for OS). Using a multivariable Cox proportional hazards model adjusted for potentially confounding prognostic factors such as age, ECOG performance status, and HER2 overexpression, it was found that the cutoff value of DpR ≥ 45% (*vs*. < 45%) was a significant predictor for OS (median 23.5 vs. 13.1 months, hazard ratio [HR] = 0.441; 95% CI = 0.204 to 0.955; Table 4).

**Table 4:** Multivariable analysis of selected demographic variables and ETS or DpR categorized by cutoff values

		Progression-free Survival			Overall Survival		
		Adjusted for Covariates			Adjusted for Covariates		
		HR	95% CI	*P*	HR	95% CI	*P*
Age	< 65	Ref			Ref		
	≥ 65	0.816	0.433 to 1.540	0.531	0.945	0.441 to 2.026	0.885
ECOG performance status	0	Ref			Ref		
	≥ 1	1.689	0.928 to 3.075	0.086	2.404	1.145 to 5.049	0.021
HER2 overexpression by IHC	2+	Ref			Ref		
	3+	0.521	0.274 to 0.993	**0.048**	0.949	0.429 to 2.100	0.897
ETS	< 30%	Ref			Ref		
	≥ 30%	0.665	0.371 to 1.193	0.171	0.687	0.332 to 1.423	0.312
Age	< 30%	Ref			Ref		
	≥ 65	0.896	0.488 to 1.626	0.720	1.025	0.488 to 2.147	0.950
ECOG performance status	0	Ref			Ref		
	≥ 1	1.582	0.861 to 2.915	0.141	2.213	1.045 to 4.675	0.038
HER2 overexpression by IHC	2+	Ref			Ref		
	3+	0.587	0.298 to 1.827	0.122	1.224	0.526 to 2.848	0.639
DpR	< 45%	Ref			Ref		
	≥ 45%	0.609	0.335 to 1.107	0.104	0.441	0.204 to 0.955	**0.038**

Abbreviations: HR, hazard ratio; CI, confidence interval; Ref, Reference; ECOG, Eastern Cooperative Oncology Group; HER2, human epidermal growth factor receptor 2; IHC, immunohistochemistry; ETS, early tumor shrinkage; DpR, depth of response

### Post-progression survival prediction using DpR and ETS

Predicting survival after progression is potentially useful for selecting patients recommended for second-line chemotherapy or further clinical trials. For this purpose, a subgroup of patients who received second-line chemotherapy (*N* = 37) were evaluated for PPS and its relation to DpR or ETS. On univariate analysis, changes in DpR were significantly correlated with PPS (Supplementary Table 4). After adjusting for age, ECOG performance status, and HER2 overexpression using IHC, DpR was the only independent predictive factor for PPS (HR = 0.844; 95% CI = 0.712 to 0.999; Supplementary Table 5). The cutoff value of 45% was a significant predictor of patients with longer PPS (*P* = 0.02; Figure 3F).

## DISCUSSION

To our knowledge, our study is among the first to evaluate DpR as a predictor of long-term outcome and to compare the predictive abilities of DpR and ETS in AGC patients. Both DpR and ETS were significant predictors for PFS and OS in AGC patients who were treated with first-line trastuzumab-containing chemotherapy.

We recognized DpR as a novel and effective measure of treatment outcome and conducted our study to compare DpR with ETS in order to determine which tumor-size metric is more predictive of prognosis. In recent large randomized phase III trials comparing cetuximab in combination with FOLFIRI as first-line treatments in colorectal cancer patients [13], the two arms did not show a difference in PFS or objective response rate, although the OS was longer in the FOLFIRI-plus-cetuximab arm. An independent radiological review suggested that the increased DpR observed in the FOLFIRI-plus-cetuximab arm may explain in part the significant OS advantage [16]. This result implies that responses to therapy might not be adequately evaluated when performed by only using RECIST; however, RECIST-independent tumor-size metrics such as DpR can better predict or categorize patients who will respond to therapy or survive longer. As DpR or ETS can easily be calculated from numbers obtained through RECIST measurements, DpR can swiftly be adopted in future clinical trials as another surrogate marker or a secondary end-point and can consequently be adopted routinely in clinical practice.

By employing Youden's *J* index, we found the optimal cutoff values to be 30% for ETS and 45% for DpR; however, only the DpR cutoff successfully classified patients according to their survival prognosis. Our identification of DpR as a dichotomous variable (45% cutoff point) makes it easily applicable to clinical practice. When adjusted for potential confounding factors, it was found that the cutoff value of DpR ≥ 45% (*vs*. < 45%) was a significant predictor for OS yet not for PFS (Table 4). This result also supports the notion of using DpR as a surrogate marker for OS.

The survival benefit of second-line chemotherapy compared to best supportive care has been demonstrated in several recent studies in patients with AGC [17–21]. Although second-line treatment now represents a standard of care in AGC, predictive factors regarding the efficacy of second-line chemotherapy are needed, especially considering the limited benefit of available therapies. Therefore, we examined whether DpR or ETS can predict PPS, as that could potentially serve as a guide for designing second-line chemotherapy or clinical trials. DpR was the only relevant factor in this case; a cutoff value of ≥ 45% identified patients with better PPS. Therefore, studies examining the efficacy of second-line therapies could be stratified according to the DpR achieved during first-line therapy. Our results of gastric cancer patients on first-line Trastuzumab with DpR ≥ 45% showing longer PPS and OS imply that a certain subgroup of patients with partial response (response rate ≥ 30%) yet without major DpR change (45%) will eventually have worse prognosis in terms of PPS or OS. For this group of patients, considering the bad prognosis of the advanced gastric cancer patients with short PPS, clinicians might consider a change to second-line chemotherapy for patients on first-line chemotherapy without improvement of cancer-related symptoms. Furthermore, the importance of DpR in predicting PPS was reported in *KRAS* wild-type colorectal cancer patients treated with cetuximab-based chemotherapy [11], implying that this notion can be applied to other tumor types and anti-tumor agents.

As a previous study revealed that TTG was one of the metrics that predicted OS in colorectal cancer patients receiving cetuximab [12], we re-conducted a multivariable analysis with DpR, including TTG as a covariate, and confirmed that DpR was a significant prognostic factor for survival outcome (Supplementary Table 6). One confounding factor was the absolute tumor burden. However, when multivariable analyses included the adjusted baseline sum of the longest diameters of target lesions (tumor burden) as a factor, DpR nevertheless remained significantly associated with OS and PFS in our study (Supplementary Table 7).

While this study revealed several valuable findings, it also had several limitations or arguments. First, this study was a retrospective study by design; thus, there may have been potential confounding factors in predicting long-term survival outcomes. Second, this study had a relatively small sample size; however, the patients included in this study were very homogeneous, which helped to minimize variability in patient prognoses. Third, as the patients in this study were treated with both trastuzumab and cytotoxic chemotherapy (XP or FP), we could not conclude that our results, which demonstrated the ability of DpR and ETS to predict prognostic outcome in HER2-positive gastric cancer patients, were attributable to the effect of trastuzumab alone. However, the proportion of patients who scored 3+ for HER2 overexpression by IHC was higher among patients with ETS ≥ 30% and DpR ≥ 45% than that of those who scored 2+ (*P* = 0.042 and *P* = 0.003, respectively; Supplementary Table 3), suggesting that DpR and ETS are good metrics for patients treated with trastuzumab. Additionally, there was no statistically significant difference between patients who received adjuvant chemotherapy after curative gastrectomy (*n* = 9) and chemo-naive patients (*n* = 52) in terms of PFS (Supplementary Figure 4). This result also supports the hypothesis that cytotoxic chemotherapy does not have a great effect on the ability of DpR and ETS to predict prognostic outcome in HER2-positive gastric cancer patients. Even if the results were attributable to cytotoxic chemotherapy, this study nevertheless highlights the importance of DpR in predicting long-term outcomes. Fourth, DpR or ETS were mainly evaluated from measurable metastatic sites, some might argue that the HER2 tests were mainly performed in primary cancer tissues and therefore DpR or ETS may not fully reflect the antitumor effect of Herceptin. However, since current clinical practice or guidelines do not have any recommendation for re-assessment of HER2 status from metastatic sites, and since previous studies reported high concordance rates of HER2 status between primary gastric cancer and paired metastatic sites [22, 23], we think that DpR or ETS would reflect anti-tumor effect of Herceptin from primary sites. Lastly, as biologically gastric cancer in the East differs from that in the West, doubts could be raised regarding the applicability of this study's results to gastric cancer patients in the West. Western countries have a much higher incidence of gastric cancer that is located in the gastroesophageal junction, and there is a higher prevalence of diffuse histology in Western gastric cancer patients [24]. HER2-positivity rates are higher in specimens from the gastroesophageal junction than in specimens from the body of the stomach and are also higher in the histologically intestinal type than in mixed or diffuse types [25]. Less than 10% of the patients in this study had gastroesophageal-junction cancer; thus, we could not generally adopt our concept of DpR and ETS in predicting long-term outcomes in gastric cancer patients treated with trastuzumab to patients in the West. However, considering that there were no differences in HER2-positivity between European and Asian countries based on the ToGA trial screening data (23.9% vs. 23.6%; *n* = 3665) and the Western patients more significantly responded to trastuzumab than the Eastern patients in the ToGA trial, we expect that DpR and ETS will be significant predictors of long-term outcomes among Western gastric cancer patients treated with trastuzumab as well.

In conclusion, our results strongly suggest that DpR is a significant predictor of long-term outcomes including PFS, OS, and PPS among AGC patients treated with trastuzumab. We suggest a cutoff value of DpR ≥ 45% to identify patients with better prognoses; this could guide clinical decision making and further identify patients likely to obtain additional benefits from trastuzumab or further chemotherapies. Moreover, DpR may be a promising and valuable endpoint in designing future clinical trials. Further validation of these results in prospective trials with larger populations and with other tumor types is warranted.

## MATERIALS AND METHODS

### Patients and assessments

A single-institution retrospective cohort analysis was performed at Yonsei Cancer Center, Seoul, Korea. The study included HER2-positive locally advanced, recurrent, or metastatic gastric cancer patients who were histologically confirmed to be inoperable and had been treated with first-line capecitabine plus cisplatin (XP) or fluorouracil plus cisplatin (FP) combined with trastuzumab. Patients with progression or death events before the first scheduled response evaluation time were excluded from the study cohort and used in the landmark analysis. Patients without measurable lesions as defined by the Response Evaluation Criteria in Solid Tumors (RECIST) version 1.1 [26] were also excluded. Electronic medical records were analyzed retrospectively in accordance with the Declaration of Helsinki. The Institutional Review Board in Severance Hospital, Seoul, Korea, reviewed and approved this study (IRB approval Number: 4-2014-1076).

Tumor samples were deemed HER2-positive if their HER2 scores were 3+ on IHC or if they stained positive for HER2 on silver *in situ* hybridization (SISH) or fluorescence *in situ* hybridization (FISH; *HER2:CEP17* ratio ≥ 2). Two independent radiologists blinded to patient data assessed target lesion responses at various time points using computed tomography (CT) according to RECIST version 1.1 every two cycles until disease progression or withdrawal. The relative change in the sum of the longest diameters of target lesions at the first response evaluation compared to baseline was defined as ETS. DpR was defined as the relative change in the sum of the target lesions’ longest diameters at their smallest attained sizes compared to baseline (TTG; time to tumor growth). PFS was defined as the interval between the first trastuzumab administration and the first documentation of progression or death; OS was calculated as the time between first trastuzumab administration and death of any cause. PPS was defined as the survival difference between OS and PFS (PPS = OS − PFS).

### Statistical analysis

Survival probabilities were estimated using the Kaplan-Meier method, and a log-rank test was used to evaluate differences between survival curves. A Cox proportional hazards regression model was used for univariate and multivariable analyses. Multivariable time-dependent concordance (C_τ_) indices and area under the curve (AUC) values for long-term outcomes were obtained from time-dependent receiver operating characteristics (TDROC) curve analyses to evaluate sensitivity and specificity [27]. Nonparametric bootstrapping with 1000 replicates was used to determine the 95% confidence intervals (CIs) of the C_τ_ indices. Youden's *J* index was calculated to determine the threshold point at which to dichotomize the patient population based on DpR or ETS in order to predict the PFS or OS [28]. All *P-values* were two-sided, and *P* < 0.05 was considered statistically significant.

SPSS version 20 was used for survival analysis via the Kaplan-Meier method. The statistical software package R version 13.0 was used to run all other analyses including TDROC and the Cox proportional hazards regression model.

## SUPPLEMENTARY MATERIALS FIGURES AND TABLES




