# Supplementary material for: Depth of response is a significant predictor for long-term outcome in advanced gastric cancer patients treated with trastuzumab
Source: Oncotarget. 2017 Mar 10;8(19):31169–79. doi: 10.18632/oncotarget.16099 (PMC5458198; doi:10.18632/oncotarget.16099)
Supplement: Supplementary file 1 [file oncotarget-08-31169-s001.pdf]

Depth of response is a significant predictor for long-term outcome in advanced gastric cancer patients treated with trastuzumab

Supplementary Materials

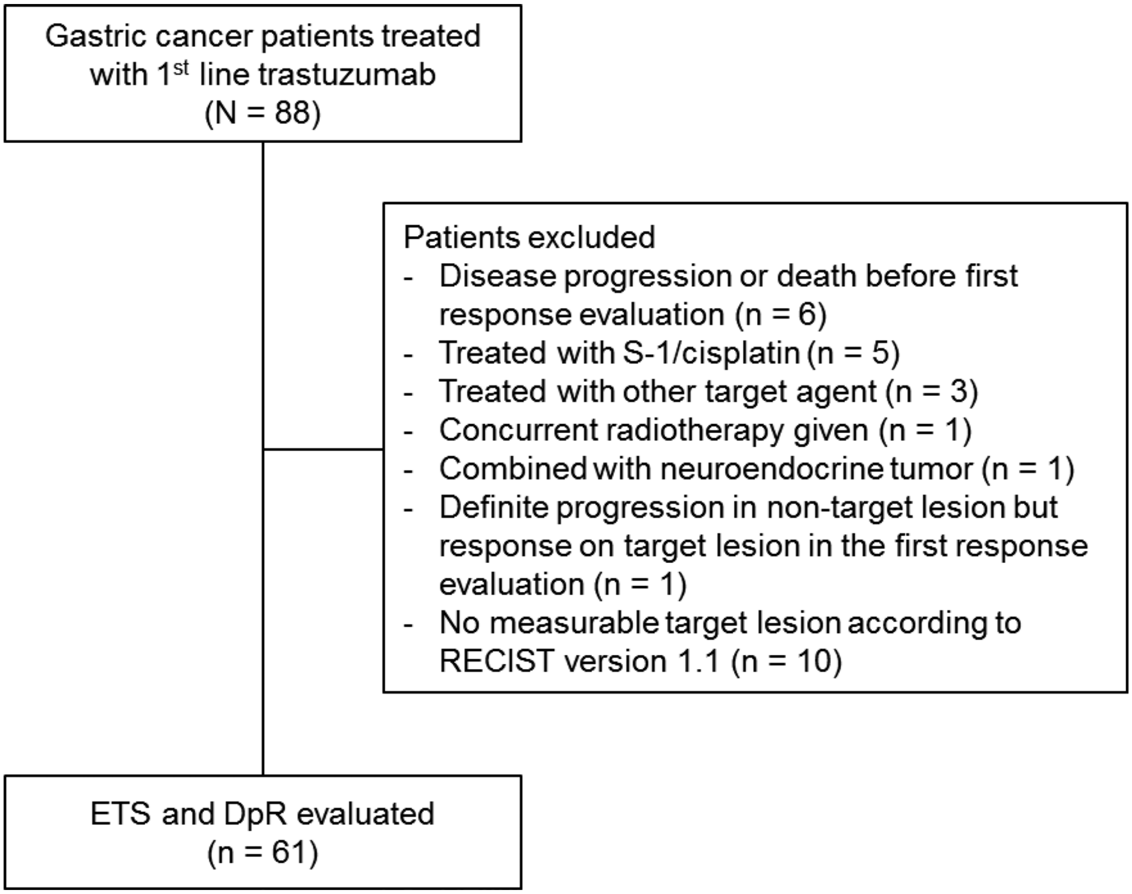

Supplementary Figure 1: Study flow diagram. RECIST, Response Evaluation Criteria in Solid Tumors; ETS, early tumor shrinkage; DpR, depth of response; PPS, Post-progression survival.

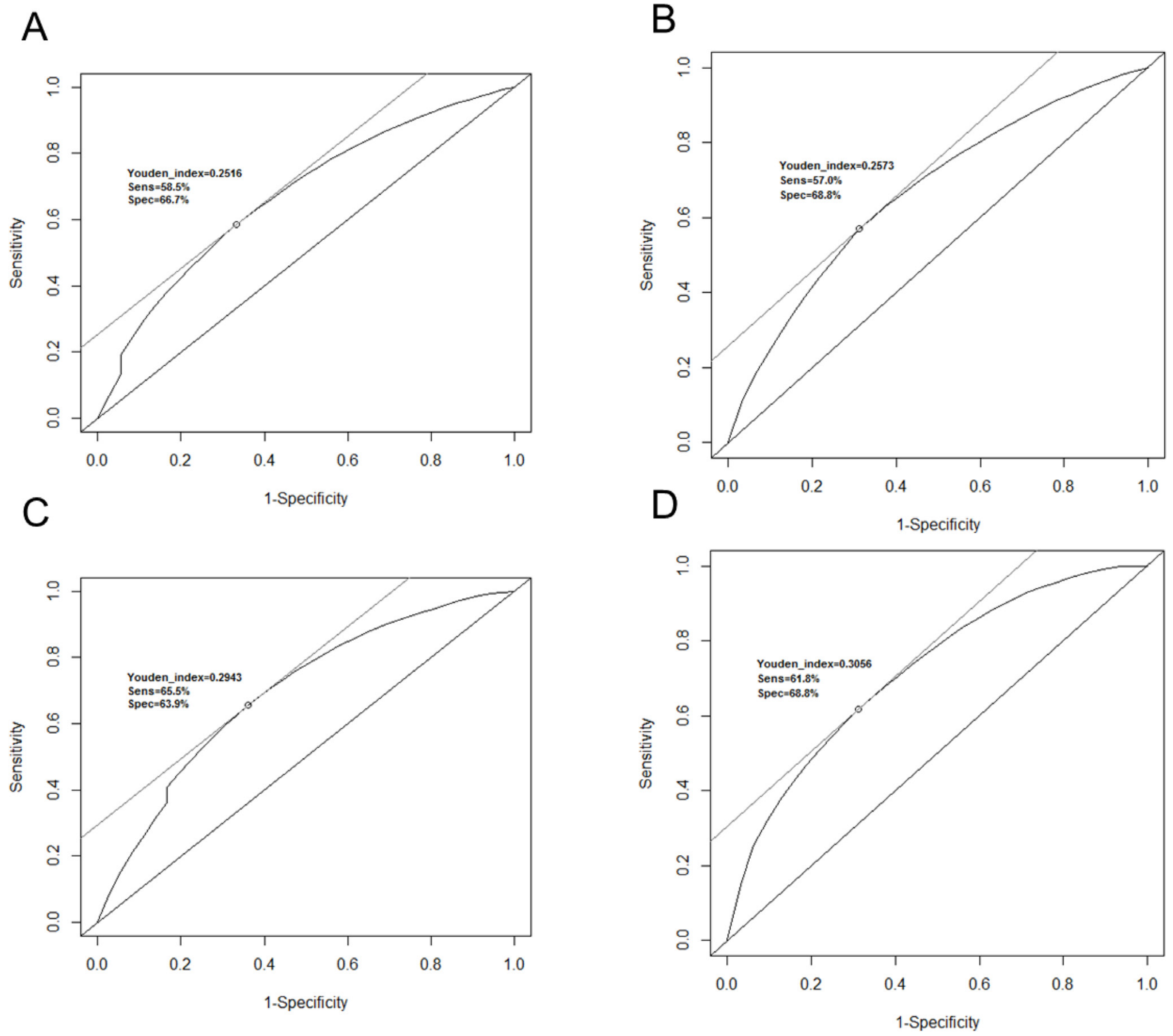

Supplementary Figure 2: ROC curves of DpR (**C, D**) and ETS (**A, B**) depicting the Youden's J index and optimal cutoff point for PFS (**A, C**) or OS (**B, D**)ETS, early tumor shrinkage; DpR, depth of response; PFS, progression-free survival; OS, overall survival.

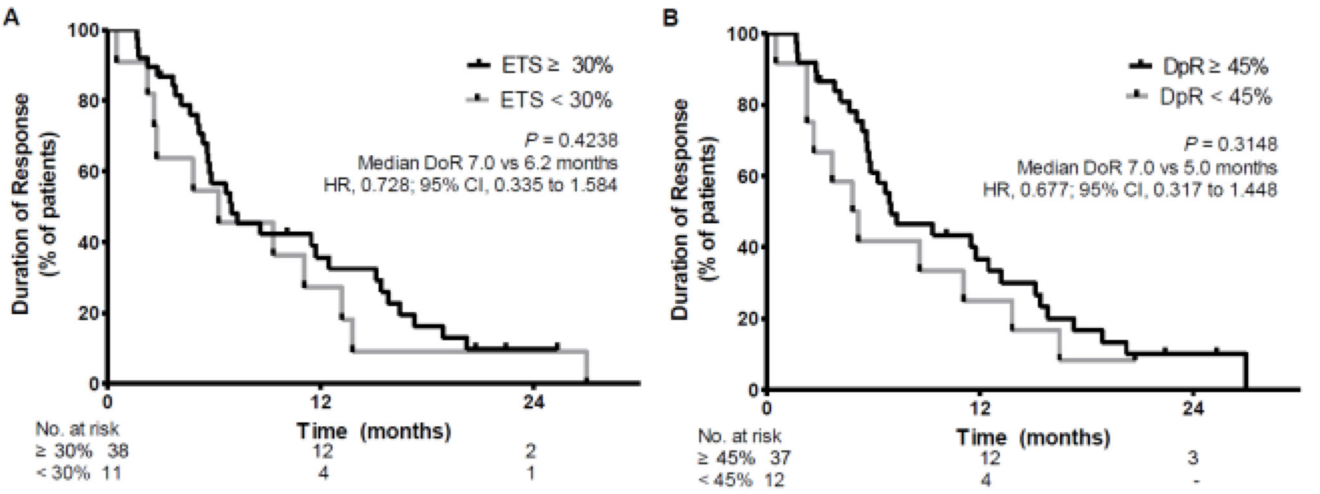

Supplementary Figure 3: Duration of Response comparing patients who reached ETS and DpR cut off values or not.

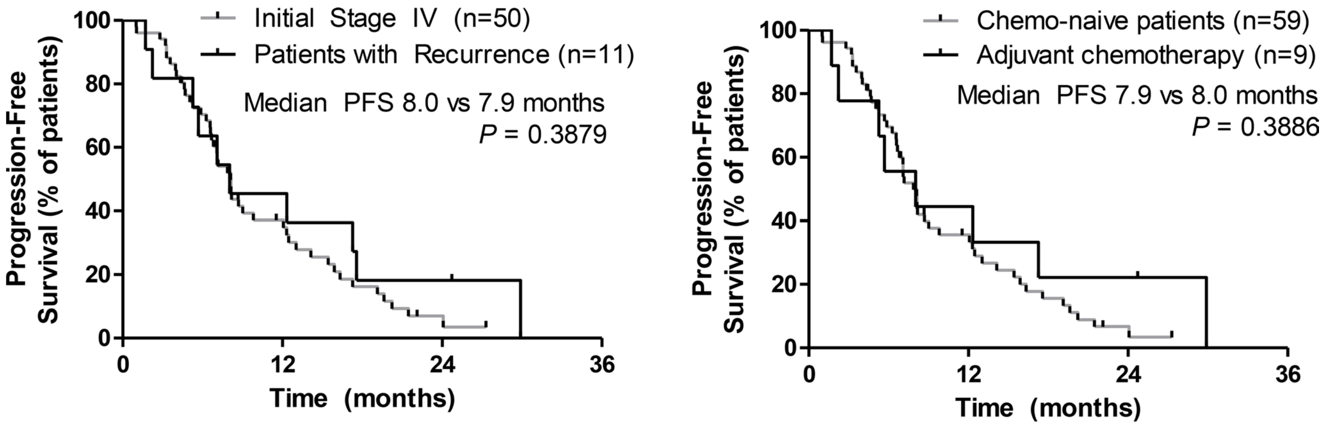

Supplementary Figure 4: Progression-free survival based on recurrence or adjuvant chemotherapy.

Supplementary Table 1: Univariate analysis of selected demographic variables for survival

	Progression-free Survival			Overall Survival		
	HR	95% CI	*P*	HR	95% CI	*P*
Sex						
Female	Ref			Ref		
Male	1.272	0.717 to 2.256	0.410	1.108	0.540 to 2.275	0.779
Age						
< 65	Ref			Ref		
≥ 65	1.001	0.573 to 1.752	0.996	1.183	0.590 to 2.373	0.635
ECOG performance status						
0	Ref			Ref		
≥ 1	1.887	1.090 to 3.267	**0.023**	2.578	1.280 to 5.192	**0.008**
HER2 overexpression by IHC						
2+	Ref			Ref		
3+	0.428	0.232 to 0.792	**0.007**	0.673	0.327 to 1.385	0.282
HER2/CEP17 ratio by SISH or FISH*						
2.0–4.69	Ref			Ref		
≥ 4.7	0.461	0.191 to 1.110	0.084	1.047	0.414 to 2.651	0.922
Metastasis						
Lymph node	0.877	0.458 to 1.678	0.692	0.764	0.354 to 1.646	0.491
Liver	1.129	0.653 to 1.952	0.664	1.508	0.768 to 2.964	0.233
Peritoneum	1.260	0.689 to 2.303	0.453	1.423	0.693 to 2.922	0.337
Lung	0.741	0.322 to 1.653	0.464	1.105	0.427 to 2.861	0.837
Bone	2.099	0.812 to 5.423	0.126	0.744	0.225 to 2.459	0.628
Chemotherapy regimen						
Herceptin + FP	Ref			Ref		
Herceptin + XP	0.787	0.369 to 1.681	0.536	1.310	0.460 to 2.861	0.613
Second Line Chemotherapy						
No	Ref			Ref		
Yes	1.607	0.898 to 2.879	0.110	0.896	0.427 to 1.881	0.772
ETS[†]	0.839	0.745 to 0.945	**0.004**	0.847	0.745 to 0.962	**0.010**
TTG	0.995	0.992 to 0.998	**0.002**	0.994	0.989 to 0.999	**0.010**
DpR[†]	0.763	0.682 to 0.853	**< 0.001**	0.804	0.723 to 0.894	**< 0.001**

[*]total 22 patients were assessed for SISH or FISH.† HR per 10% tumor size decrease Abbreviations : HR, hazard ratio; CI, confidence interval; Ref, Reference; ECOG, Eastern Cooperative Oncology Group; HER2, human epidermal growth factor receptor 2; IHC, immunohistochemistry; CEP17, chromosome 17 centromere; SISH, silver in situ hybridization; FISH, fluoroscence in situ hybridization; FP, 5-FU+Cisplatin; XP, Xeloda+Cisplatin; ETS, early tumor shringkage; TTG, time to tumor growth; DpR, depth of response.

Supplementary Table 2: Multivariable analysis of selected demographic variables

	Progression-free Survival			Overall Survival		
	Adjusted for Covariates			Adjusted for Covariates		
	**HR**	**95% CI**	***P***	**HR**	**95% CI**	***P***
Age						
< 65	Ref			Ref		
≥ 65	0.678	0.354 to 1.300	0.242	1.041	0.502 to 2.160	0.915
Sex						
Female	Ref			Ref		
Male	1.539	0.833 to 2.842	0.168	1.433	0.661 to 3.107	0.362
HER2 overexpression by IHC						
2+	Ref			Ref		
3+	0.490	0.259 to 0.931	**0.029**	1.017	0.455 to 2.272	0.970
ECOG performance status						
0	Ref			Ref		
≥ 1	1.883	0.999 to 3.547	0.050	2.573	1.215 to 5.450	**0.014**
Regimen						
Herceptin + FP	Ref			Ref		
Herceptin + XP	0.545	0.236 to 1.262	0.157	1.517	0.503 to 4.577	0.459
ETS[†]	0.850	0.754 to 0.959	**0.008**	0.854	0.733 to 0.996	**0.044**
Age						
< 65	Ref			Ref		
≥ 65	0.715	0.381 to 1.340	0.295	1.159	0.559 to 2.402	0.692
Sex						
Female	Ref			Ref		
Male	1.340	0.718 to 2.504	0.358	1.277	0.5778 to 2.825	0.545
HER2 overexpression by IHC						
2+	Ref			Ref		
3+	0.579	0.302 to 1.110	0.100	1.543	0.666 to 3.578	0.312
ECOG performance status						
0	Ref			Ref		
≥ 1	1.479	0.766 to 2.854	0.244	2.155	0.990 to 4.691	**0.053**
Regimen						
Herceptin + FP	Ref			Ref		
Herceptin + XP	0.593	0.259 to 1.356	0.215	2.345	0.720 to 7.646	0.157
DpR[†]	0.787	0.698 to 0.888	**< 0.0001**	0.765	0.659 to 0.888	**0.0004**

[†]HR per 10% tumor size decrease.

Abbreviations : HR, hazard ratio; CI, confidence interval; Ref, Reference; ECOG, Eastern Cooperative Oncology Group; HER2, human epidermal growth factor receptor 2; IHC, immunohistochemistry; FP, 5FU+Cisplatin; XP, Capecitabine+Cisplatin; ETS, early tumor shringkage; TTG, time to tumor growth; DpR, depth of response.

Supplementary Table 3: Comparison of clinicopathologic features of patients categorized by cutoff value on ETS or DpR (*N* = 61). See Supplementary_Table_3

Supplementary Table 4: Univariate analysis of selected demographic variables of patients who had undergone second chemotherapy (*N* = 37) and their relation to post progression survival

	Post Progression Survival		
	**HR**	**95% CI**	***P***
Sex			
Female	Ref		
Male	0.872	0.601 to 2.106	0.760
Age			
< 65	Ref		
≥ 65	0.716	0.295 to 1.735	0.459
ECOG performance status			
0	Ref		
≥ 1	1.704	0.755 to 3.846	0.199
HER2 overexpression by IHC			
2+	Ref		
3+	1.102	0.470 to 2.580	0.824
HER2/CEP17 ratio by SISH or FISH*			
2.0–4.69	Ref		
≥ 4.7	1.442	0.493 to 4.218	0.504
Metastasis			
Lymph node	0.520	0.222 to 1.220	0.133
Liver	2.060	0.916 to 4.631	0.080
Peritoneum	0.863	0.356 to 2.091	0.744
Lung	1.635	0.552 to 4.838	0.375
Bone	0.540	0.159 to 1.840	0.325
Chemotherapy regimen			
Herceptin + FP	Ref		
Herceptin + XP	2.180	0.469 to 3.806	0.588
ETS[†]	1.073	0.810 to 1.072	0.325
TTG	0.995	0.989 to 1.001	0.130
DpR[†]	0.884	0.784 to 0.998	**0.046**

[*]total 17 patients were assessed for SISH or FISH.

[†]HR per 10% tumor size decrease.

Abbreviations : HR, hazard ratio; CI, confidence interval; Ref, Reference; ECOG, Eastern Cooperative Oncology Group; HER2, human epidermal growth factor receptor 2; IHC, immunohistochemistry; CEP17, chromosome 17 centromere; SISH, silver in situ hybridization; FISH, fluoroscence in situ hybridization; FP, 5-FU+Cisplatin; XP, Xeloda+Cisplatin; ETS, early tumor shringkage; TTG, time to tumor growth; DpR, deepness of response.

Supplementary Table 5: Multivariable analysis of selected demographic variables of patients who had undergone second chemotherapy ($N = 37$) and their relation to post progression survival

	Post Progression Survival		
	Adjusted for Covariates		
	**HR**	**95% CI**	***P***
Age			
< 65	Ref		
≥ 65	0.733	0.298 to 1.801	0.498
ECOG performance status			
0	Ref		
≥ 1	1.591	0.679 to 3.728	0.268
HER2 overexpression by IHC			
2+	Ref		
3+	1.760	0.618 to 5.008	0.290
ETS†	0.910	0.763 to 1.084	0.289
Age			
< 65	Ref		
≥ 65	1.002	0.379 to 2.654	0.997
ECOG performance status			
0	Ref		
≥ 1	1.276	0.498 to 3.271	0.611
HER2 overexpression by IHC			
2+	Ref		
3+	2.977	0.960 to 9.236	0.059
TTG	0.997	0.989 to 1.006	0.459
DpR†	0.844	0.712 to 0.999	**0.049**

†HR per 10% tumor size decrease.

Abbreviations : HR, hazard ratio; CI, confidence interval; Ref, Reference; ECOG, Eastern Cooperative Oncology Group; HER2, human epidermal growth factor receptor 2; IHC, immunohistochemistry; ETS, early tumor shringkage; TTG, time to tumor growth; DpR, depth of response.

Supplementary Table 6: Multivariable analysis of selected demographic variables with time to tumor growth

	Progression-free Survival			Overall Survival		
	Adjusted for Covariates			Adjusted for Covariates		
	**HR**	**95% CI**	***P***	**HR**	**95% CI**	***P***
Age						
< 65	Ref			Ref		
≥ 65	1.141	0.477 to 1.611	0.672	1.336	0.617 to 2.896	0.462
ECOG performance status						
0	Ref			Ref		
≥ 1	1.188	0.641 to 2.202	0.584	1.575	0.719 to 3.451	0.256
HER2 overexpression by IHC						
2+	Ref			Ref		
3+	0.704	0.370 to 2.342	0.287	1.801	0.756 to 4.316	0.183
TTG	0.998	0.995 to 1.000	0.074	0.996	0.990 to 1.001	0.095
DpR†	0.823	0.730 to 0.927	**0.001**	0.840	0.731 to 0,965	**0.014**

†HR per 10% tumor size decrease.
Abbreviations : HR, hazard ratio; CI, confidence interval; Ref, Reference; ECOG, Eastern Cooperative Oncology Group; HER2, human epidermal growth factor receptor 2; IHC, immunohistochemistry; ETS, early tumor shringkage; TTG, time to tumor growth; DpR, depth of response.

Supplementary Table 7: Multivariable analysis of DpR and survival outcomes after adjusting tumor burden

	Progression-free Survival			Overall Survival		
	Adjusted for Covariates			Adjusted for Covariates		
	HR	95% CI	*P*	HR	95% CI	*P*
Age						
< 65	Ref			Ref		
≥ 65	0.739	0.398 to 1.375	0.340	0.792	0.353 to 1.778	0.572
ECOG performance status						
0	Ref			Ref		
≥ 1	1.385	0.759 to 2.528	0.288	1.882	0.884 to 4.009	0.101
HER2 overexpression by IHC						
2+	Ref			Ref		
3+	0.601	0.318 to 1.137	0.118	1.353	0.589 to 3.108	0.477
Baseline Sum of LD	1.002	0.998 to 1.006	0.409	1.006	1.001 to 1.011	**0.026**
DpR[†]	0.802	0.710 to 0.906	**0.000**	0.837	0.731 to 0.958	**0.010**

[†]HR per 10% tumor size decrease

Abbreviations : HR, hazard ratio; CI, confidence interval; Ref, Reference; ECOG, Eastern Cooperative Oncology Group; HER2, human epidermal growth factor receptor 2; IHC, immunohistochemistry; LD, long diameters; DpR, depth of response
